# The Use and Effectiveness of an Online Diagnostic Support System for Blood Film Interpretation: Comparative Observational Study

**DOI:** 10.2196/20815

**Published:** 2021-08-09

**Authors:** Claire Hutchinson, Michelle Brereton, Julie Adams, Barbara De La Salle, Jon Sims, Keith Hyde, Richard Chasty, Rachel Brown, Karen Rees-Unwin, John Burthem

**Affiliations:** 1 Medicine, Dentistry and Human Sciences Faculty of Health University of Plymouth Plymouth United Kingdom; 2 University Hospitals Plymouth NHS Trust Plymouth United Kingdom; 3 Manchester Foundation Trust Manchester United Kingdom; 4 UK NEQAS Haematology Watford United Kingdom; 5 The Christie NHS Foundation Trust Manchester United Kingdom; 6 Division of Cancer Sciences University of Manchester Manchester United Kingdom

**Keywords:** blood cell morphology, decision support, external quality assessment in hematology, diagnosis, digital morphology, morphology education

## Abstract

**Background:**

The recognition and interpretation of abnormal blood cell morphology is often the first step in diagnosing underlying serious systemic illness or leukemia. Supporting the staff who interpret blood film morphology is therefore essential for a safe laboratory service. This paper describes an open-access, web-based decision support tool, developed by the authors to support morphological diagnosis, arising from earlier studies identifying mechanisms of error in blood film reporting. The effectiveness of this intervention was assessed using the unique resource offered by the online digital morphology Continuing Professional Development scheme (DM scheme) offered by the UK National External Quality Assessment Service for Haematology, with more than 3000 registered users. This allowed the effectiveness of decision support to be tested within a defined user group, each of whom viewed and interpreted the morphology of identical digital blood films.

**Objective:**

The primary objective of the study was to test the effectiveness of the decision support system in supporting users to identify and interpret abnormal morphological features. The secondary objective was to determine the pattern and frequency of use of the system for different case types, and to determine how users perceived the support in terms of their confidence in decision-making.

**Methods:**

This was a comparative study of identical blood films evaluated either with or without decision support. Selected earlier cases from the DM scheme were rereleased as new cases but with decision support made available; this allowed a comparison of data sets for identical cases with or without decision support. To address the primary objectives, the study used quantitative evaluation and statistical comparisons of the identification and interpretation of morphological features between the two different case releases. To address the secondary objective, the use of decision support was assessed using web analytical tools, while a questionnaire was used to assess user perceptions of the system.

**Results:**

Cases evaluated with the aid of decision support had significantly improved accuracy of identification for relevant morphological features (mean improvement 9.8%) and the interpretation of those features (mean improvement 11%). The improvement was particularly significant for cases with higher complexity or for rarer diagnoses. Analysis of website usage demonstrated a high frequency of access for web pages relevant to each case (mean 9298 for each case, range 2661-24,276). Users reported that the decision support website increased their confidence for feature identification (4.8/5) and interpretation (4.3/5), both within the context of training (4.6/5) and also in their wider laboratory practice (4.4/5).

**Conclusions:**

The findings of this study demonstrate that directed online decision support for blood morphology evaluation improves accuracy and confidence in the context of educational evaluation of digital films, with effectiveness potentially extending to wider laboratory use.

## Introduction

### Background

The morphological assessment of blood cells in different diseases is a “gatekeeper” investigation for abnormal findings identified using automated blood analyzers [1]. Important decisions are made following all levels of blood film review as morphologists must identify abnormal features, and also interpret their significance [2,3]. Those decisions range from the content of comments concerning the appearances of blood cells, through to urgent direct communication of important findings that might have an immediate impact on patient care [3,4]. Support for morphological decision-making is therefore vital, and traditionally this has been provided through training at the microscope, supplemented by the use of books and posters and the ability to refer decisions to experienced colleagues. However, providing good quality local support at all times has become challenging, as changing shift patterns and service models can reduce available support, and also impact on the cognitive processes required for accurate assessment [5,6]. Responses have included the increased use of computer-based support including semiautomated image recognition systems [7-9], as well as the increasing use of online information. Although internet-prepared resources can be very valuable, it is vital that they have accurate content and an accessible platform of delivery and are open access. It is also essential that the provision of diagnostic support is linked to objective assessment of the value of that support [10].

### Prior Work

The authors of this paper have more than 15 years of experience in delivering online training through various media, including provision to developing countries [11,12] and through the UK National External Quality Assessment Service (UK NEQAS) Haematology Digital Morphology Continuing Professional Development scheme (DM CPD scheme) established in 2005 [13]. This digital scheme grew to meet the need for professional development in morphological diagnosis [14] and has more than 3000 registered users in the UK and internationally. The system has a good correlation with glass slide morphology, and has been well received by users [13]. In a recent study, the authors used data from this CPD scheme to address the mechanisms of error by morphologists interpreting blood film appearances, aiming to identify interventions that could improve diagnostic outcome [15]. The data from that study identified distinct patterns of error and suggested that diagnostic accuracy could be improved and mistakes could be reduced through the provision of information that specifically addressed these major sources of error.

### Approach

This paper describes a web-based system for education and decision support, made available through desktop or mobile devices to support the morphological assessment of blood films. The system was developed to address the varied approaches required to assess cases of different types and complexity. In this paper, we assess the design considerations for the decision support website based on our previous work. Using the unique resources offered by the UK NEQAS Haematology DM CPD scheme, we then evaluate the user experience and effectiveness of the system in improving the outcomes of blood film assessment.

## Methods

### The Digital Morphology CPD Scheme

The Digital Morphology CPD scheme has been described in earlier publications [14]. In brief, more than 50 continuous microscopic fields were captured using Zeiss Axio Imager.M1 with a Zeiss Plan-Apochromat 63×/1.4 oil lens, then stitched using the photomerge function of Adobe Photoshop CS4 (version 11.02; Adobe Inc) and optimized for sharpness (unsharp mask function, 1 pixel up to 100%), lighting, and color balance (curves function). The final image was reviewed to ensure accurate and sufficient representation of the diagnostic features for each case, then uploaded to either Digital Slidebox (SlidePath, Leica Biosystems) or EQATE UK NEQAS Haematology Online (Certus Technology Associates Ltd). Each system provided equivalent functionality for image viewing and data submission (representative images from the EQATE system are shown in Multimedia Appendix 1). Users of the UK NEQAS DM scheme viewed the images via web browser, submitting their morphological selections and preferred diagnosis. Feedback was made using online forms with free-text responses or user grading (1-5; Google Forms, Google). The 5 cases that were rereleased used identical films. The cases were given new narratives and feedback available after case completion, and participants had no direct access to the previous release of these cases.

### The Decision Support System

This was developed by the authors, and approved for use with the UK NEQAS DM CPD scheme by the Morphology Special Advisory Group of UK NEQAS General Haematology. Content was reviewed by the author team with feedback from users. The approach is outlined in the Results section; images used were “typical cell forms” (drawn using the range of functions of Photoshop CS4) or photographed images. Supporting written information was developed using the authors’ experience, together with published sources.

The system was delivered using a MediaWiki platform (version 1.28.3; MediaWiki) to allow compatibility across a range of mobile devices and desktop systems. Settings prevent user edits. Consistent with the Wiki Foundation principles, readers do not impart any personal information. Links to the system from the CPD scheme were provided with each case, and the system does not restrict access by individual users. Page use data was obtained through host analytics (1&1 Ionos) to solely provide the date, number of page access episodes, and geographical location (country); this was not linked to any retained personally identifiable information.

### Data Analysis and Statistics

The participant responses for each DM CPD case (fully anonymized) were exported to Excel (Microsoft Corp). The data analyzed comprised the following: the number of participants, self-reported experience level, and for each film and user response, the morphological features selected as being present and the preferred diagnosis or diagnostic comment (free text). All free-text responses were then coded according to diagnosis by the authors to facilitate statistical comparison. Statistical comparison was performed using GraphPad Prism (version 7.03; GraphPad Software Inc), with details given in figure legends.

## Results

### Design Principles and Implementation

**Figure 1:**
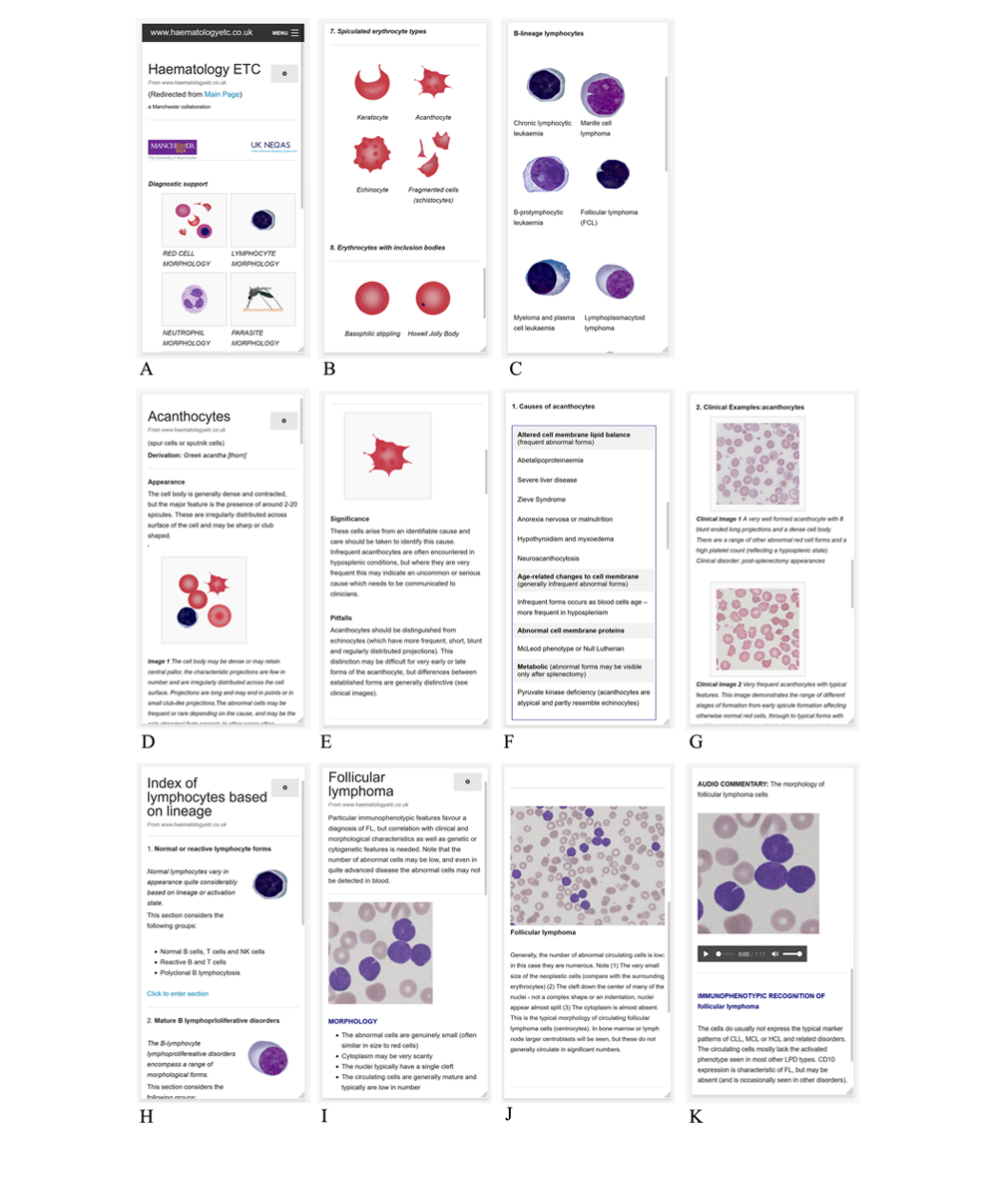
Representative screenshot details from the decision support app. Panels A-C show details of image-driven indexing: (A) main index, (B) red blood cell index, and (C) lymphocyte index. Panels D-G show details from red blood cell pages (acanthocyte morphology page) showing the following: (D) detailed cell description, (E) significance and pitfalls in diagnosis, (F) causes, and (G) clinical examples. Panels H-K show details from lymphocyte pages: (H) functional index, (I) follicular lymphoma main description, (J) clinical context, and (K) audio commentaries.

A decision support system was developed and made available to users through desktop or mobile devices. Of 320 participants responding to our survey, the majority used a desktop system (n=294, 92%), although laptops (31%), tablets (n=30, 9%), and mobile phones (n=30, 9%) were frequently used, either in addition to or instead of a desktop device. The system has image-driven menus that show typical morphological forms (Figure 1A-C) linked to more detailed interpretive and confirmatory support through text and clinical images. For each cell type, information was presented that reflects the different requirements of interpretation: red blood cell sections centered particularly on identifying the range of different appearances that may occur together on a blood film, with supporting information focusing on the significance of the combinations of features found (Figure 1D-G). The white blood cell sections focused predominantly on the evaluation of cytological features of individual cells (Figure 1H-K). The full working decision support site has free open access [16].

### The Use and Outcomes of the Decision Support System

**Figure 2:**
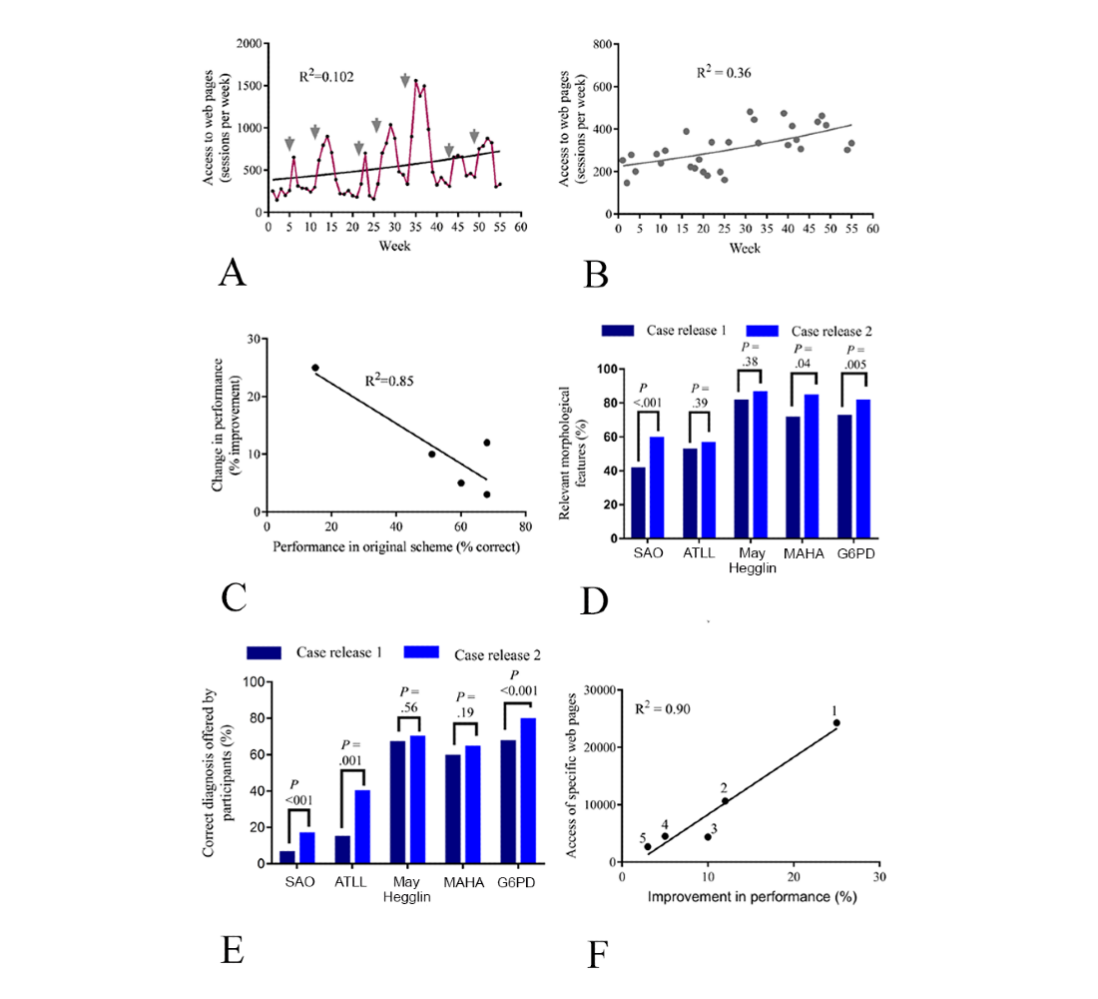
Access to decision support and accuracy of Continuing Professional Development completion. (A) web activity over time (red line) with trend line (black line) indicating changing weekly access numbers; each new case release is indicated by an arrow. (B) The same data excluding access associated with case availability, showing an approximate doubling of access by UK users and weak to moderate correlation. (C) A high number of web visits in the most recent case release was very strongly correlated with increasing case difficulty (as indicated by the percentage of correct responses when first released; *R*^2^=0.85). Numbers refer to individual cases as assigned in Table 1. (D) Accuracy of reporting of the major morphological features for each case in the two separate releases, showing a consistently improved accuracy when decision support was available. (E) The number of participants accurately identifying the abnormal findings was increased in the more recent release, particularly in the more difficult cases. (F) The level of improvement in outcome was strongly correlated with increased access of relevant pages of the decision support tool (numbers refer to individual cases as assigned in Table 1). Statistical testing for (D) and (E): chi-square test with Yates correction. ATLL: adult T-cell leukemia lymphoma; G6PD: glucose-6-phosphate dehydrogenase; MAHA: microangiopathic hemolytic anemia; SAO: Southeast Asian ovalocytosis.

**Figure 3:**
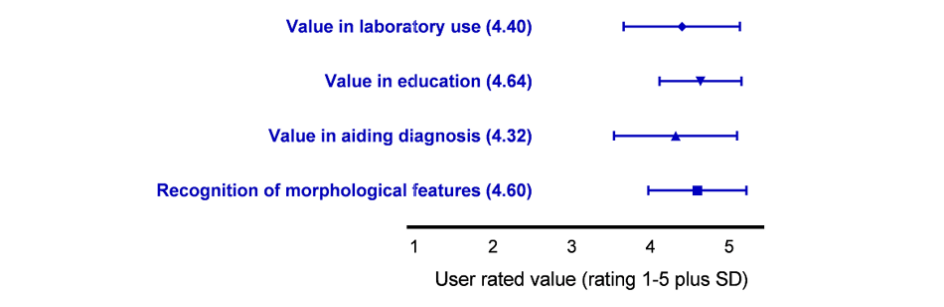
Participant interaction with and perception of the system. Users were asked their view of the value of the system in the contexts indicated. Users rated the system 1-5, where 1 was the lowest score and 5 was the highest score (data shows mean and SD of a user sample of 200). Users were asked the following questions: (1) Do you find HaematologyETC useful in helping you to identify morphological features in CPD cases? (2) Do you find HaematologyETC useful in helping you to reach a diagnosis in CPD cases? (3) How would you rate HaematologyETC as an educational tool? (4) Do you think HaematologyETC would be useful in assisting morphology reporting in your laboratory?

Use of the decision support system was optional, and participants were also free to access any other reference source. To map use of the system, analytic software was used to record access to pages from each country, without recording personal identifiable data. This showed that, when compared with the reference period, there was a significant increased use of the site during the 3-week period when each case was available (Figure 2A). The average number of UK individuals accessing the main site menu each week during each reference period (when no CPD case was available) was 932, compared to a mean of 2359 per week when cases were active (mean difference 1427, *P*=.02, n=23,000 observations, Wilcoxon test of paired data). The mean number of relevant pages accessed during each case release was 9298 (range 2661-24,276). The mean number of participants who submitted entries to the CPD scheme for each case was 1022, suggesting that a substantial proportion of participants visited the site on at least one occasion and viewed multiple relevant pages. Additionally, a weak to moderate trend for increased general use of the site was also noted, with an overall trend to approximately double over 12 months when time points reflecting “live” CPD assessments were excluded (Figure 2B). When the use of decision support was compared with the degree of difficulty of the case—as indicated by the proportion of incorrect responses (% incorrect) when the case was first released—it was found that the use of decision support was very strongly correlated with the difficulty of the case (*R*^2^=0.85; Figure 2C). Finally, users were directly asked their perception of the system regarding different aspects of use. For users responding to the survey request (n=230), most reported increased confidence in identification of features (4.6/5) and diagnosis (4.4/5). The system was valued highly in the context of education (4.7/5) and in routine laboratory usage (4.4/5; Figure 3).

Overall, these data reflect widespread use of the decision support system to support CPD scheme activity, with selective use of those pages according to the perception of case difficulty, as well as increased general use of the system outside of the scheme. We therefore went on to assess whether the decision support tool led to improved accuracy of morphological assessment and interpretation. To achieve this, 5 digital images that had previously been used in the CPD scheme were rereleased in identical form, but now with users given access to the decision support system. To avoid these cases being recalled by participants, no case was used if it had been released in the previous 4 years, and the mean interval was 5.4 years between the two release dates (Table 1). The number of registered users was found to be increased in the more recent period and access to the cases was increased overall from 5375 to 6663 participants (24%); however, the experience of users in each assessment period was comparable (the self-reported proportion of qualified staff reporting blood films regularly for a representative case was similar between the two periods [536/713 participants, 75% compared with 976/1354, 72%]). This analysis showed that the availability of the decision support tool for participants in the more recent cases led to a more accurate recognition of the morphological forms present in the digital image (based on those features selected by participants as most characteristic of the blood smear). The improvement varied between cases, with a mean of 9.8% (n=2902 users in initial 5 case releases and n=3922 in recent 5 case releases, range of improvement 4%-18%, *P*=.02, Student *t* test of matched pairs; Figure 2D). Furthermore, the diagnostic accuracy was also consistently improved in the more recent cases when decision support was available, with an overall improvement of 11% (n=2902 users in initial 5 case releases and n=3922 in recent 5 case releases, range of improvement 3%-25%, *P*=.02, Wilcoxon test of paired *t* test data), and the greatest improvement seen in “more difficult” cases where diagnostic accuracy in the initial case release was lower (Figure 2E). These more difficult cases were also associated with much higher rates of use of the decision support tool (as reflected by web access of pages relevant to the case diagnosis; Figure 2F).

Overall, analysis showed that the accuracy of diagnosis was significantly better across the full case set (total participants contributing diagnoses n=6824, mean accuracy 55% versus 44%, *P*=.02, Student *t* test for paired data).

**Table 1:** Case number, description, and participant data.

Case number	Diagnosis	First release date	Participants, first release, n	Participants, second release, n	Change, %
1	Southeast Asian ovalocytosis with Epstein-Barr virus infection (Southeast Asian ovalocytosis/glandular fever)	2014	991	1392	40
2	Adult T-cell leukemia lymphoma (ATLL)	2015	1102	1418	28
3	May-Hegglin anomaly (May-Hegglin)	2013	1104	1271	15
4	Microangiopathic hemolytic anemia (MAHA)	2015	1277	1544	21
5	Oxidative hemolysis in glucose-6-phosphate dehydrogenase deficiency (G6PD)	2011	901	1092	21

### Effect on Decision-making in Complex Morphological Cases

**Figure 4:**
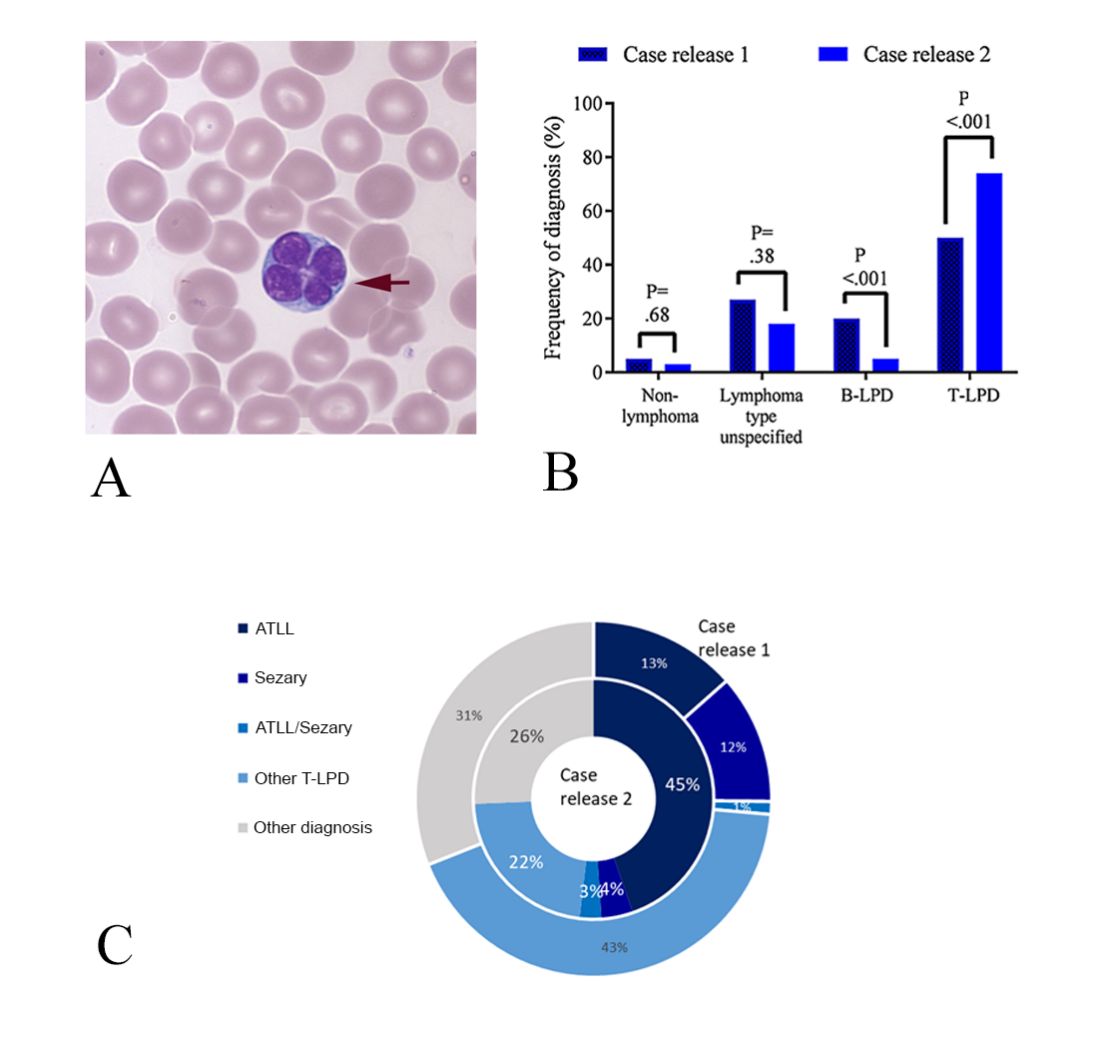
Detailed analysis of the case with ATLL. (A) Typical morphological features of the case, demonstrating a particularly characteristic large abnormal lymphocyte showing a lobulated "clover leaf" nucleus (arrowed). (B) A greater proportion of participants identified the diagnosis as lymphoma; of these, a significantly greater proportion identified the T-cell nature of the disease. (C) Pie chart comparing diagnostic choice for white blood cell features in release 1 and release 2, showing significantly improved rates of specific diagnosis and of diagnostic accuracy in the more recent release (release 2). Statistical testing for (B): chi-square test with Yates correction. ATLL: adult T-cell leukemia lymphoma.

The case of adult T-cell leukemia lymphoma (ATLL) demonstrated the challenge of detailed morphological evaluation and interpretation of abnormalities when there were small numbers of abnormal cells (Figure 4A). The case required participants first to recognize that the lymphoid cell population was abnormal, then to make a detailed morphological assessment of the cells present, and ascribe a likely diagnosis based on that assessment. This case was difficult for participants when first released, with only around 16% making the correct diagnosis. When rereleased, the case attracted particularly frequent use of the decision support website, with more than 18,000 relevant pages accessed by participants, of which more than 3700 were specific for the diagnosis of ATLL. This high use of support was associated with an improved accuracy in the different stages of the diagnostic process; in the initial release, 829 participants answered the case, with 1282 participants in the more recent release. Although participants identified the lymphocytosis with equal frequency, those in the more recent case release group were more likely to identify the cells as neoplastic (n=615, 48% versus n=328, 40%, *P*<.001, chi-square test, Fisher exact test) and were also significantly more likely to suggest that the cells represented a neoplastic T-cell disorder (934/1282 participants versus 414/829, 73% versus 50%; *P*<.001, chi-square test with Yates correction; Figure 4B). This was associated with a significantly greater likelihood of ascribing a precise diagnosis to the case (rather than a generic diagnosis of T-cell lymphoproliferative disorder) as well as a much higher recognition that the diagnosis was ATLL (513/1282, 45% versus 108/829, 13%; *P*<.001, chi-square test with Yates correction; Figure 4C). These findings suggest that support for the detailed cytological evaluation of rare cell types was widely used, and that this resulted in improved accuracy in the morphological evaluation of uncommon disorders.

### Effect on Decision-making in the Presence of Multiple Abnormal Features

For those diagnoses where a range of abnormal morphological features are present, interpretation requires strategies that depend on the identification, classification, and prioritization of the abnormal features. The accuracy of these strategies depends very much on the skill and knowledge of the morphologist, and may result in unhelpful simplification processes such as elimination bias (where features are ignored or assigned inappropriately low significance for diagnosis) or framing bias (where features are considered to fit a single preferred diagnosis, irrespective of whether this is the most likely interpretation).

**Figure 5:**
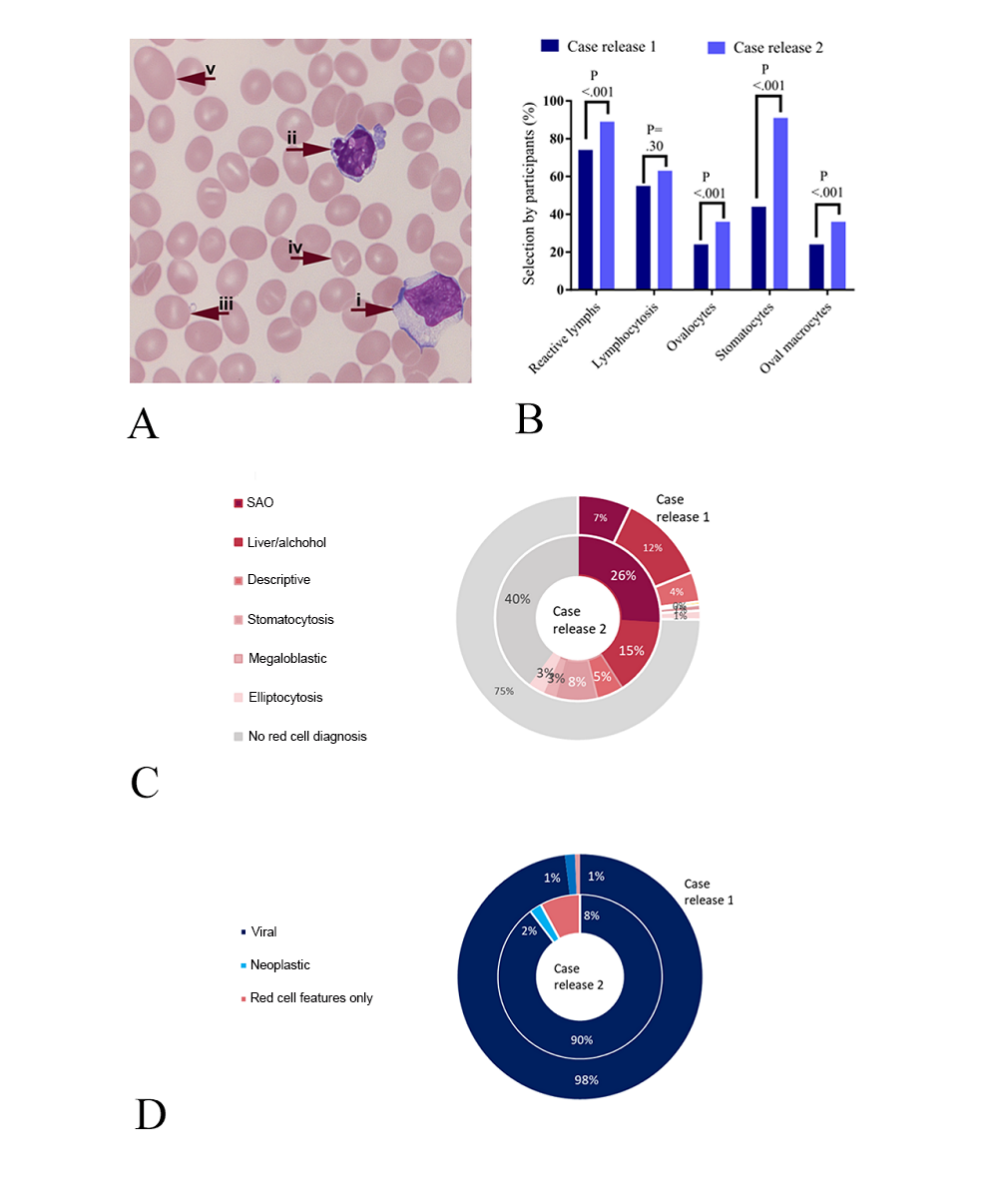
Detailed analysis of the case with SAO and EBV infection. (A) Typical morphological features of the case, demonstrating (i) a large activated lymphocyte, (ii) an apoptotic lymphocyte, and (iii) the erythroid abnormalities including stomatocytes, some of which have (iv) complex stoma and (v) very large oval cells. (B) Accuracy of morphological evaluation by participants, showing a higher percent selection of all relevant morphological features of the case, particularly the reporting of stomatocytes. (C) Pie chart comparing diagnostic choice for red blood cell features in release 1 and release 2, showing improved accuracy in the second release. (D) Pie chart comparing diagnostic choice for white blood cell features in release 1 and release 2, showing a trend for better accuracy in the first release. Statistical testing for (B): chi-square test with Yates correction. EBV: Epstein-Barr virus; SAO: Southeast Asian ovalocytosis.

Consistent with this, the most complex case in this set combined the red blood cell disorder Southeast Asian ovalocytosis (SAO) with an accompanying Epstein-Barr virus infection, leading to distinctive but complex morphology (Figure 5A). This case proved difficult for participants when first released, with a tendency for errors related to simplification strategies; in the initial release, the marked red blood cell abnormalities were all reported relatively infrequently, particularly the diagnostically important stomatocytes (Figure 5B), and red blood cell diagnoses were offered by only 245 of 991 (25%) participants. The correct interpretation of SAO was made by just 69 of 991 participants (7%) in the initial release (Figure 5C). In contrast, the features of viral illness were well recognized (reported and correctly interpreted by 961/991 participants, 97%; Figure 5D). When the case was rereleased, the decision support tool was heavily used, with more than 11,000 views of pages relevant to the case, and this was associated with a more accurate identification of abnormal morphological features (Figure 5B), with the overall consideration of red blood cell disorders in the final diagnosis increased to 841/1402 participants (60%; *P*<.001; Figure 5C) and the correct diagnosis of SAO given by 365/1402 participants, (26%; *P*<.001, chi-square test with Yates correction). Interestingly, the increased awareness of the red blood cell features led to indications that some participants used a framing bias, attempting to unify the white blood cell and red blood cell features into a single diagnosis of liver disease or malignancy (Figure 5D). A small proportion showed elimination bias, as they simply omitted any white blood cell diagnosis at all. Overall, the reduced rate of diagnosis of viral disorder was not significant (*P*=.19, chi-square test with Yates correction).

## Discussion

### Principal Results

In this study, digital blood films provided within the online UK NEQAS Haematology DM CPD scheme were used to evaluate the effectiveness of a new decision support system developed by the authors to support morphological assessment in hematology. This analysis revealed that use of the decision support site was associated with a significantly improved accuracy of reporting for both morphological feature identification and the interpretation of those features. That information, together with web page analytic data and direct user feedback, has shown that the system was widely used by CPD participants and improved the performance and confidence of users.

The detailed analysis of the two most difficult cases was particularly useful to evaluate the effectiveness of support. For white blood cell types, the system focused on providing detailed help in assessing cytology of individual cell types to support recognition of characteristic features that allow discrimination between different cell forms. This help was very highly used in the assessment of the uncommon lymphoproliferative disorder ATLL, and this use was associated with a very substantial improvement both in recognizing the unusual (T-cell) nature of the disorder and in suggesting the correct diagnosis (ATLL). For red blood cell diagnoses, the aim of the system was (as for white blood cells) to provide help in recognizing abnormal forms, and additionally focused on identifying pitfalls in cell recognition and showing how combinations of features could be used to make a diagnosis. In the case of SAO, the participants more frequently reported those relevant red blood cell features present and also showed considerable improvement in suggesting the correct diagnosis (SAO).

### Possible Limitations of the Study

The decision support was evaluated using an online education system and compared present users examining DM CPD cases with the historical performance of users assessing the same case. We recognize that the study design therefore contains potentially confounding factors related to the separate time points. In particular, users in the current period were likely to have increased familiarity with technology and online resources and were more likely to have access to better technical resources, screen quality, and download speed [17,18]. The study partly addresses these limitations, showing that irrespective of other factors the decision support pages were frequently accessed at times when CPD cases were available for assessment. In addition, the web pages accessed corresponded closely to the subject of the case, consistent with directed use of the system by participants. Furthermore, there was evidence of selective use of pages according to case difficulty, with a marked correlation between the number of times relevant pages were accessed and the difficulty of the case (as indicated by performance in the earlier period).

It is also worth noting that the system does not address all sources of error. The case of SAO also had reactive (“viral”) lymphocytes, which were very widely reported in the earlier release; interestingly, the improved awareness of red blood cell features was associated with a small reduction in those reporting the likely viral disorder. These errors were similar to the “heuristic errors” observed in our previous study of cases with a highly complex group of features [15] and may reflect familiar sources of bias in complex cases, such as “anchoring” or “elimination” bias, where morphologists focused only on red blood cell features.

### Context and Prior Work

Providing effective support for morphological diagnosis requires an understanding of the process; the complexity and number of cells present on a blood film means that (consciously or unconsciously) morphologists employ approaches to reduce the complexity of the evaluation to allow a timely conclusion [19,20]. Briefly, the process has two phases. The first phase is “perception,” a process where the morphologist perceives whether the observed appearances differ from an expectation of normality; this is a rapid and largely subconscious process [21], whose effectiveness depends significantly on the experience of the observer [22]. The second phase employs the skills of “recollection and analysis” to evaluate particular findings in detail [23], requiring the active application of knowledge through techniques that simplify analysis, such as classification (allowing abnormal cells to be considered as classes or groups rather than as separate entities), prioritization (to focus on those elements that are considered most important to diagnosis), and elimination (to remove from consideration those elements considered unimportant to diagnosis). These techniques all require an appropriate knowledge base for accurate outcomes; when incorrectly performed, the process can lead to errors of various types (reviewed in [15,24,25]).

The support system in this paper describes an approach to providing effective “bench side” diagnostic support for the analytical phase of blood film evaluation by providing a rapid and accessible information source available at the time of evaluation focusing on the central processes of cell recognition, classification, prioritization, and interpretation. A digital platform was selected based on the advantages offered by such a system in the context of morphology. First, digital platforms enable the uploading and displaying of many images, as well as the ability to select and magnify features or to drive links or visual menu systems. Second, digital platforms are accessible to all users and are accessed through computers or mobile devices that can be freely available at the point of need. Third, this digital approach offers a flexibility that can be iteratively modified to suit user needs or changing information; this platform can be linked to other resources and websites that provide supporting or detailed information. It is important to reflect that global health resources should offer accessibility across geographical barriers; in this case, the adoption of a MediaWiki platform and free access to all users facilitates wide use. It is recognized though that language barriers often remain. However, while the decision support system developed by the authors is written solely using English language, there are positive features for future development. First, the use of a MediaWiki platform allows access to the tools developed to facilitate easier page translation for Wikipedia and related applications. These tools support potential future collaborative development of the system in different language formats. Second, the use of image-driven menus within the system was intended to support easier navigation for those not fully familiar with morphological descriptions, but this may also enable the use of the system by a wider international group.

### Conclusions

In this paper, we have demonstrated the effectiveness of this specifically designed online tool to improve the performance of morphologists in the setting of an online CPD system. However, the broader question is whether there is an expanded role for its use in general diagnosis within laboratories. The internet is gaining wider penetration in all areas of society; the public is increasingly using and familiar with internet resources, often accessed by mobile devices. Online diagnosis support can be used either at the microscope or as an adjuvant to computer-assisted diagnostic systems. The flexibility of a web-based approach may also be extended, particularly to support fast-evolving areas such as molecular diagnosis. Finally, the system may have particular value in developing countries where internet access via mobile devices may become a major point of access for teaching, training, or other services, and where low-cost flexible support can provide wider benefit if problems of adoption can be overcome [12,26].

## Appendix

Multimedia Appendix 1 The EQATE system. (A) The menu system in the left panel allows users to select information or provide responses to the case; the panel can be expanded by users as required to provide a larger view of the image, which can be expanded or contracted to allow higher magnification viewing. (B) The full image field in the right panel shows around 50 images taken at high magnification using an oil immersion lens allowing maximum resolution of detail, while the left panel shows the narrative that guides users through the major features of the film following completion of the case. (C) Image viewed at high power (equivalent to magnification at the time of image acquisition). (D) Additional features of the case are shown at highest magnification with an accompanying annotation following completion of the case.

## Abbreviations

ATLL: adult T-cell leukemia lymphoma
CPD: Continuing Professional Development
DM: Digital Morphology
SAO: Southeast Asian ovalocytosis
UK NEQAS: UK National External Quality Assessment Service for Haematology
