# Supplementary figures and images for: The Use and Effectiveness of an Online Diagnostic Support System for Blood Film Interpretation: Comparative Observational Study
Source: J Med Internet Res. 2021 Aug 9;23(8):e20815. doi: 10.2196/20815 (PMC8386359; doi:10.2196/20815)

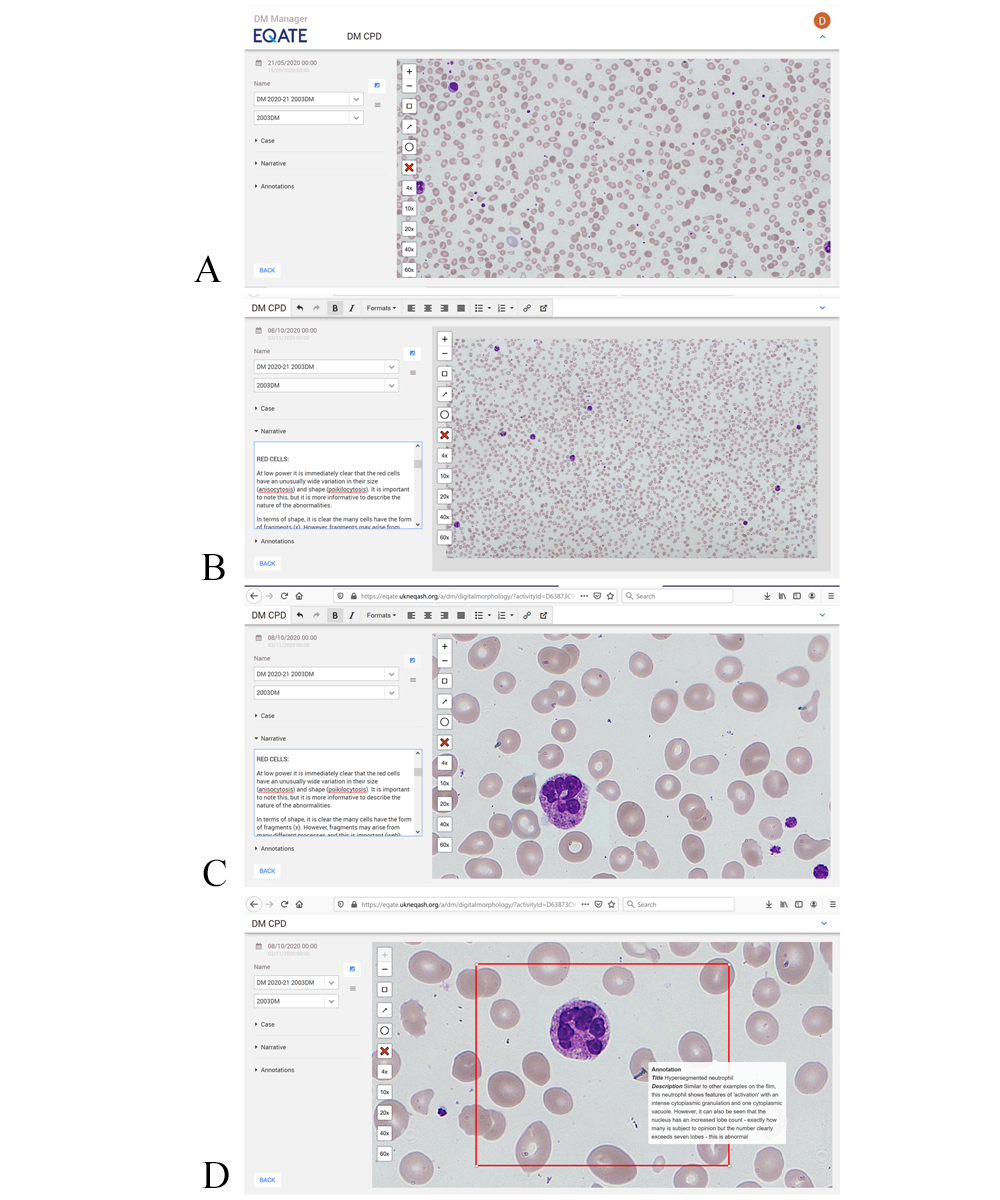

Supplement: Multimedia Appendix 1 [file jmir_v23i8e20815_app1.png]
